# Characterization of circulating miRNA signature in water buffaloes (*Bubalus bubalis*) during *Brucella abortus* infection and evaluation as potential biomarkers for non-invasive diagnosis in vaginal fluid

**DOI:** 10.1038/s41598-018-38365-x

**Published:** 2019-02-13

**Authors:** Cristina Lecchi, Carlotta Catozzi, Valentina Zamarian, Gaia Poggi, Giorgia Borriello, Alessandra Martucciello, Domenico Vecchio, Esterina DeCarlo, Giorgio Galiero, Fabrizio Ceciliani

**Affiliations:** 1 Università degli Studi di Milano, Dipartimento di Medicina Veterinaria, Milano, 20133 Italy; 2 Istituto Zooprofilattico Sperimentale del Mezzogiorno, Portici, 80055 Italy; 3 Centro di Referenza Nazionale sull’Igiene e le Tecnologie dell’Allevamento e delle Produzioni Bufaline, Salerno, 84132 Italy; 4 Diabetes Research Institute (DRI), San Raffaele Scientific Institute, Milano, 20132 Italy

## Abstract

Brucellosis is an infectious disease caused by bacteria from the *Brucella* genus that can be transmitted to humans through contact with infected animals or contaminated animal products. Brucellosis also causes financial losses in animal production. Ruminants are highly susceptible to brucellosis, and the causative agent water buffaloes (*Bubalus bubalis*) is *Brucella abortus*. Circulating microRNAs (miRNAs) are cropping up as promising biomarkers for several infectious diseases. The goals of this study were to characterize the serum miRNA signature associated with brucellosis in water buffaloes and investigate the miRNAs’ potential use as biomarkers in vaginal fluids. Next Generation Sequencing was used to assess miRNA expression profiles in *Brucella*-positive and *Brucella*-negative blood sera; dysregulated miRNAs in blood serum and vaginal fluids were validated using RT-qPCR. ROC curves were generated to evaluate the diagnostic value of miRNAs for *Brucella*. GO and KEGG pathway enrichment analyses were exploited to investigate the biological functions of dysregulated miRNAs. The results showed that 20 miRNAs were modulated, of which, 12 were upregulated and 8 were downregulated. These findings were corroborated by RT-qPCR, and ROC curves indicated that the miRNAs can serve as potential biomarkers for *Brucella*. GO and KEGG pathway analyses pointed out that some of these miRNAs are related to immune response and apoptosis. These results provided an overview of miRNA expression profiles and highlighted potential biomarkers for *Brucella* infection in water buffaloes. We also demonstrated the potential of vaginal fluids in studies involving microRNA detection. Further functional and mechanistic studies of these miRNAs may improve our understanding of the biological processes involved in *Brucella* infection and host immune response.

## Introduction

Brucellosis is one of the most important zoonotic diseases in ruminants. The aetiological agent is *Brucella*, a gram-negative, facultative, intracellular pathogen^1,2^. The favoured reproductive niche of *Brucella* is the intracellular milieu of macrophages, dendritic cells and placental trophoblasts^3^. Brucellosis is a zoonosis of considerable importance to public health with more than 500,000 new human infections being estimated annually. Brucellosis also causes financial losses in animal production^4,5^. Routine screening and animal vaccinations have led to Brucellosis disappearing in western regions, although it remains endemic in developing regions such as the Middle East, Asia, Africa and South America and in some areas of Italy^6^. *Brucella* is transmitted to humans by consuming raw milk or after direct contact with infected animals. The infectious course of brucellosis is divided into three phases, each marked by distinct bacteriological, clinical and pathological profiles: (i) onset of infection; (ii) the acute phase during which clinical, haematological and pathological symptoms are first observed; and (iii) the chronic phase, characterized by intermittent clinical symptoms and evident pathological signs^2^. Ruminants are highly susceptible to brucellosis; small and large ruminants are preferentially infected by *B. melitensis* and *B. abortus*, respectively. In most areas examined thus far, namely, South America^7,8^, Pakistan^9^, Italy^10^ and Africa^11^, the main causative agent in water buffaloes is *B. abortus* biovar 1. In pregnant females, the bacterium invades the placenta, and subsequently the foetus, prompting abortion mainly during the last third of the pregnancy^12,13^. Nonpregnant animals, still shedding the bacteria through secretions, may be asymptomatic with no evident clinical or pathological signs^14^. *Brucella* infections must be diagnosed early to control disease spreading. *B. abortus* and *B. melitensis* ruminant brucellosis are diagnosed based on bacteriological and immunological tests, the latter being routinely used in control, eradication and surveillance programmes^15,16^. Serological tests are used to initially diagnose brucellosis, but the results can be negative, even when the bacterium is present, particularly during the early disease phases. Thoroughly understanding *Brucella* biology and identifying novel biomarkers are essential for diagnosis and prophylaxis protocols. MicroRNAs (miRNAs) are small noncoding RNA that regulate gene expression posttranscriptionally. They play pivotal roles in cellular homeostasis, and their expression is dysregulated during stress conditions, disorders and diseases^17^. MicroRNA are involved in pathogen-host interactions^18^ and are stable in body fluids, from which they can be easily extracted^19^. Consequently, miRNAs are promising biomarkers for diagnosing several diseases and stress disorders in both humans^20,21^ and animals^22–24^. Changes in miRNA expression patterns have been observed in association with infectious diseases^25–27^ and as reactions to specific stresses such as thermal stress^28^. *Brucella* has also been shown to modulate *in vitro* expression of miRNAs involved in host immune responses^29–31^.

*Brucella* infection reduces fertility by inducing abortion as well as suppurative placentitis^32^. Since no information has been reported on circulating miRNAs during *Brucella* infection in water buffaloes (*Bubalus bubalis*), the present study aimed to (a) assess miRNA expression profiles in the blood sera of water buffaloes infected by *B. abortus*; (b) extract and measure miRNA expressions in vaginal fluid during *B. abortus* infection; (c) determine whether miRNAs can be used as biomarkers to assess brucellosis; and (d) integrate miRNAs to their target genes and relative biological processes.

## Results

### Identifying differentially expressed serum microRNAs during *B. abortus* infection by miRNA sequencing

Serum miRNAs were sequenced to determine the differential miRNA profiles of *B. abortus*-infected and healthy buffaloes. A total of 469 miRNAs were identified, of which, 20 showed significantly altered expression in seropositive animals compared with seronegative animals. In seropositive animals, the expressions of 12 miRNAs were upregulated 1.8- to 3.7-fold, while 8 miRNAs were downregulated 1.8- to 6.7-fold (*P* ≤ 0.05) (Table 1).

**Table 1:** Differentially expressed miRNAs in the serum of seropositive buffaloes compared with seronegative buffaloes by sequencing.

	MiRNA	Fold Change	*P* Val
UP	let-7f	2.6	0.003
UP	miR-126-5p	2.1	0.044
UP	let-7i	1.8	0.07
UP	miR-138	5.7	0.022
UP	miR-143	2.4	0.045
UP	miR-146b	3.7	0.033
UP	miR-151-3p	1.9	0.01
UP	miR-191	2.1	0.014
UP	miR-215	2.5	0.007
UP	miR-381	5.3	0.037
UP	miR-92a	1.9	0.021
UP	miR-92b	2.1	0.019
DOWN	miR-133a	−6.7	0.003
DOWN	miR-127	−3.2	0.044
DOWN	miR-150	−6.7	0.001
DOWN	miR-221	−2.7	0.017
DOWN	miR-30e-5p	−1.8	0.034
DOWN	miR-30d	−2.1	0.012
DOWN	miR-320a	−2.5	0.045
DOWN	mir-339b	−2.5	0.045

### Validating microRNA sequencing results and quantifying differentially expressed miRNAs in blood serum and vaginal fluid samples

**Figure 1:**
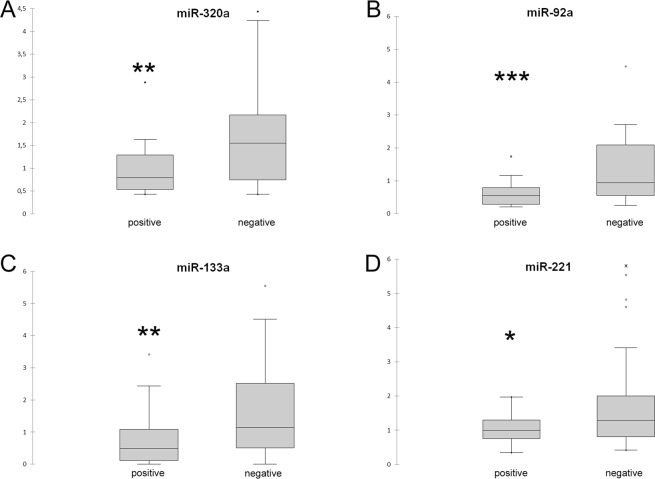
Box plots of DE-miRNAs in the blood serum. Significance was declared at *P < 0.05, **P < 0.01 and ***P < 0.001. Black lines inside the boxes mark the medians. Whiskers indicate variability outside the upper and lower quartiles.

**Figure 2:**
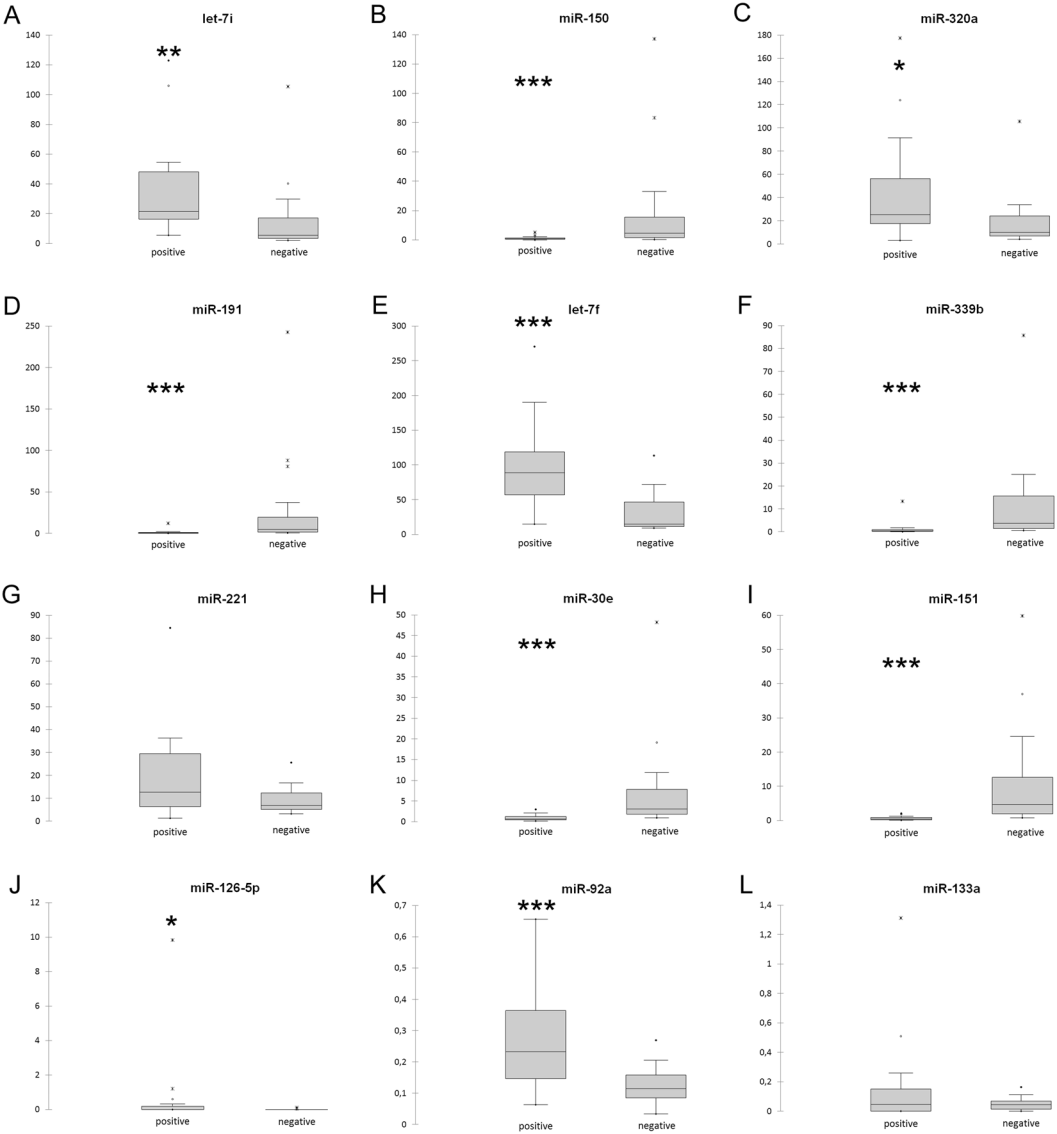
Box plots of the DE-miRNAs in the vaginal fluid. Significance was declared at *P < 0.05, **P < 0.01 and ***P < 0.001. Black lines inside the boxes mark the medians. Whiskers indicate variability outside the upper and lower quartiles.

qPCR validation was performed on the 30 sequenced samples and a separate independent cohort of 30 buffaloes. To validate the sequencing results, 12 differentially expressed (DE)-miRNAs were selected, and their relative abundance was quantified using RT-qPCR. MiRNA levels were normalized to that of cel-miR-39, an artificial spike-in which was used as an internal control. The selected miRNA targets were detected in all blood serum and vaginal fluid samples. RT-qPCR results for the blood serum demonstrated that the levels of four miRNAs (miR-320a, miR-133a, miR-92a, and miR-221) were significantly downregulated in seropositive buffaloes (Fig. 1). An analysis of 12 selected miRNAs from the vaginal fluid demonstrated that 10 miRNAs (miR-let-7i, miR-150, miR-320a, miR-191, miR-let-7f, miR-339b, miR-30e, miR-151, miR-126-5p, and miR-92a) were significantly differentially expressed between *Brucella*-positive and negative buffaloes. Figure 2 presents an overview of these results. DE-miRNA levels were unaffected by the animals’ oestrus phases (linear regression, *P* > 0.05).

### Assessing the diagnostic value of DE-miRNAs during *B. abortus* infection

**Figure 3:**
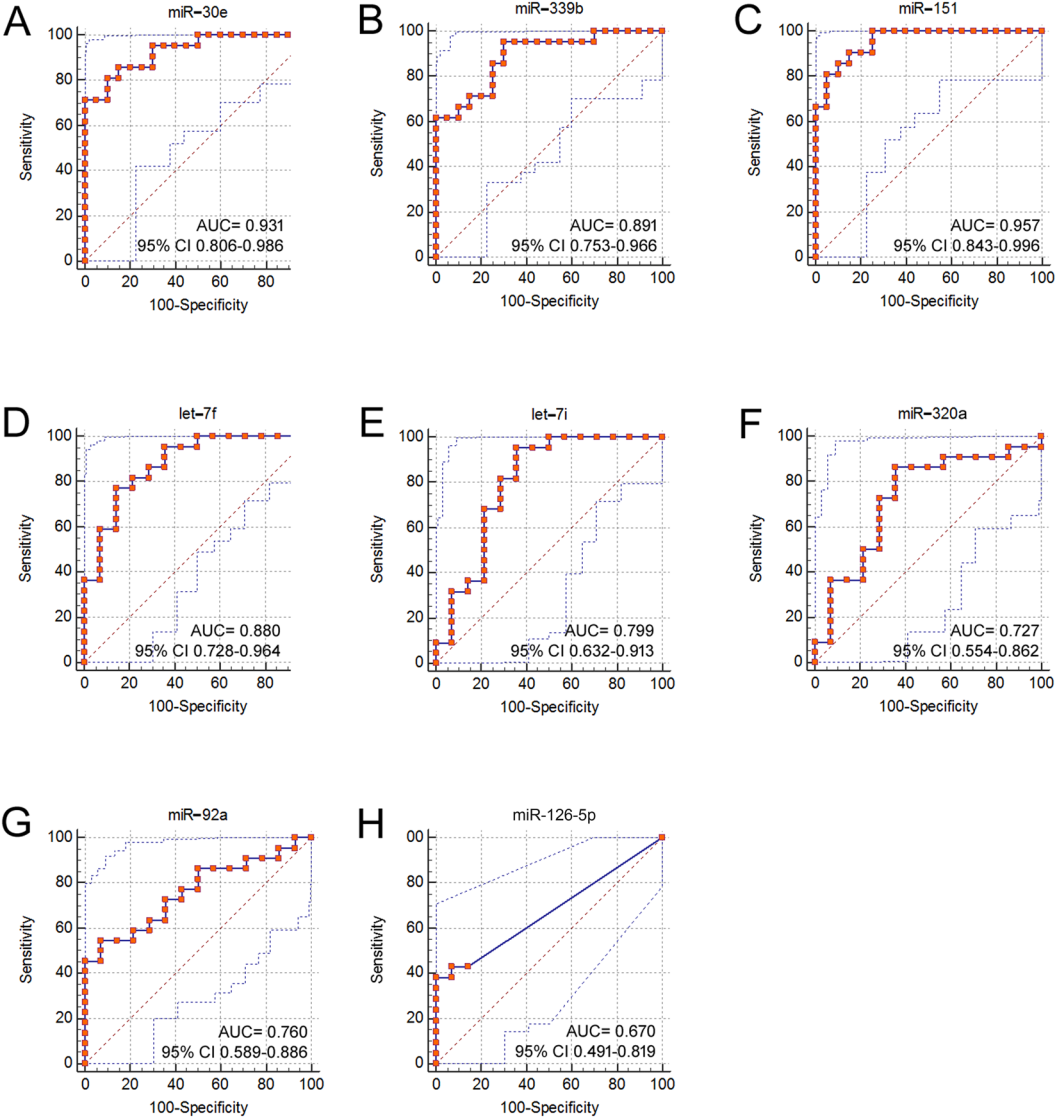
Receiver-operator characteristic (ROC) curve analysis of DE-miRNAs in the vaginal fluid. AUC, area under the curve; CI, confidence interval.

To analyse the diagnostic value of DE-miRNAs in the blood serum and vaginal fluid, ROC curves were analysed, and the associated area under the curve (AUC) was used to confirm the diagnostic potency of each miRNA. The ROC was analysed as previously reported^22^. Table 2 summarizes the diagnostic performance of each DE-miRNA and shows combinations of some DE-miRNAs. The AUC was fair for blood serum miR-320a and miR-92a and poor for blood serum miR-133a and miR-221 (Supplemental Material 1). The AUC was excellent for vaginal fluid miR-151 and miR-30e, with calculations of 0.957 and 0.931, respectively; good for miR-let-7f, miR-339b, miR-150 and miR-191 (AUC ≥ 0,799); fair for miR-let-7i, miR-92a and miR-320a; and poor for miR-126-5p (Fig. 3). To test potential collinearity, a Spearman correlational analysis was performed on the vaginal fluid miRNAs with excellent and good AUC values, suggesting that relative concentrations of miR-151, miR-339b, miR-150, miR-191, and miR-30e are positively correlated with each other (data not shown).

**Table 2:** Sensitivity, specificity, and area under the curve (AUC) for DE-miRNAs in the blood serum and vaginal fluid. MiRNAs combined in the vaginal fluid include miR-let-7f, miR-151, miR-30e, miR-191, miR-150 and miR-339b.

	MiRNA	AUC	95% CI	*P Value*	Cut-Off	Sensitivity	Specificity
BLOOD SERUM	miR-320a	0.736	0.603–0-844	0.0005	1.33	84	65.62
	miR-92a	0.749	0.616–0.854	0.0001	0.82	80	65.62
	miR-133a	0.693	0.556–0.808	0.0069	1.08	76	56.25
	miR-221	0.661	0.524–0.781	0.026	1.25	72	56.25
	miR-92a, miR-320a	0.753	0.620–0.857	0.0001	0.90	80	56.25
VAGINAL FLUID	miR-126-5p	0.67	0.491–0.819	0.0142	0.04	42.86	92.86
	miR-92a	0.76	0.589–0.886	0.0011	0.099	86.36	50
	miR-320	0.727	0.554–0.862	0.014	11.12	86.36	64.29
	miR-let7f	0.88	0.728–0.964	<0.0001	25.13	95.45	64.29
	miR-let7i	0.799	0.632–0.913	0.0007	7.57	95.45	64.29
	miR-151	0.957	0.843–0.996	<0.0001	2.0936	100	75
	miR-30e	0.931	0.806–0.986	<0.0001	2.133	95.24	70
	miR-339	0.89	0.753–0.966	<0.0001	1.85617	95.25	70
	miR-150	0.826	0.676–0.926	<0.0001	3.31	95.24	55
	miR-191	0.898	0.763–0.970	<0.0001	2.09	95.24	70
	miRNAs combination	0.88	0.742–0.959	<0.0001	0.023	94.45	85

**Figure 4:**
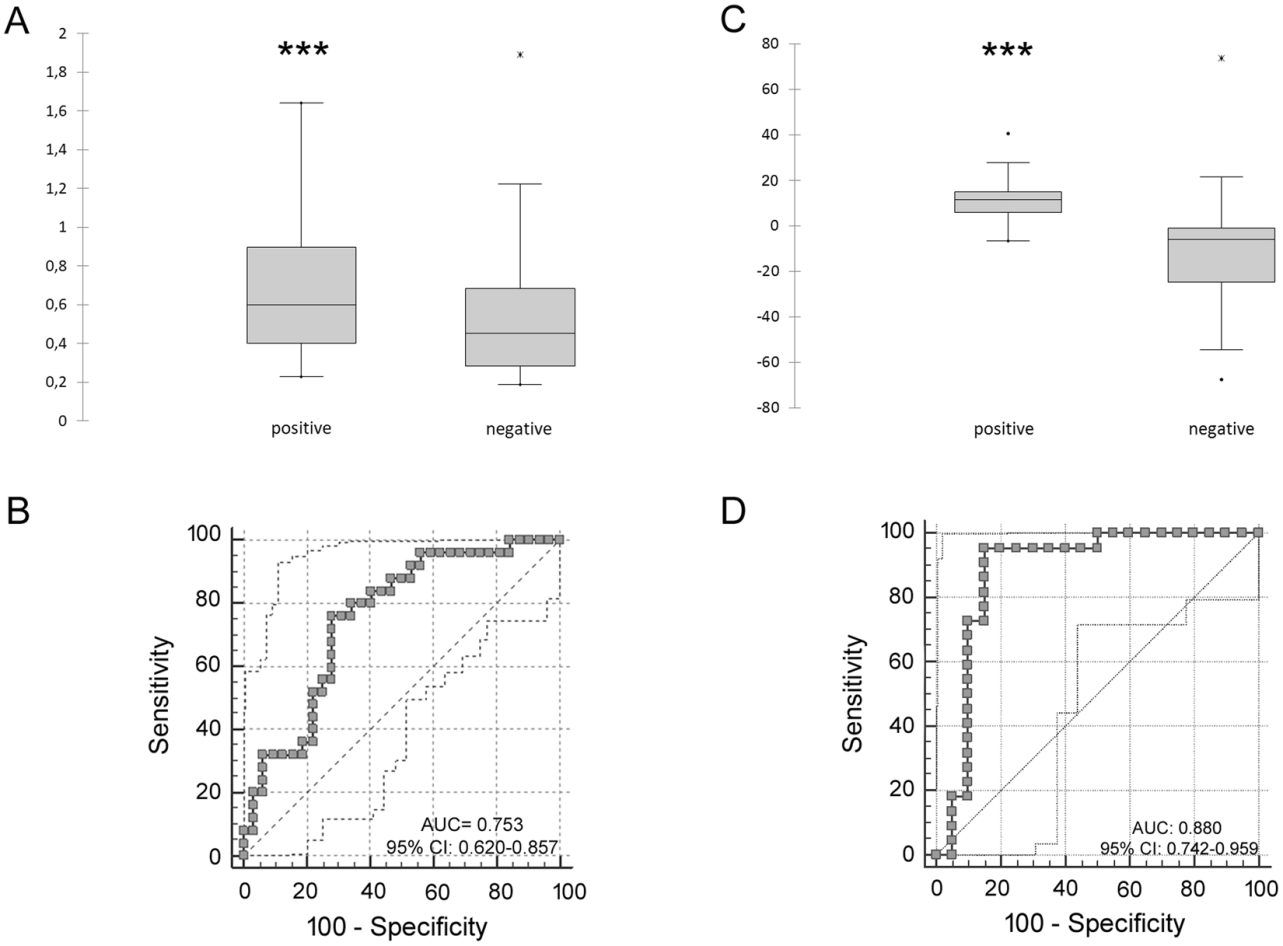
Average expression of the DE-miRNAs in the blood serum and vaginal fluid. Weighted average relative quantification (RQ) values of DE-miRNAs in the blood serum (**A**) and vaginal fluid (**C**). ROC curve analysis, constructed using the logit model, for DE-miRNAs in the blood serum (**B**) and vaginal fluid (**D**). AUC, area under the curve; CI, confidence interval. Black lines mark the medians. ***P < 0.0001.

**Figure 5:**
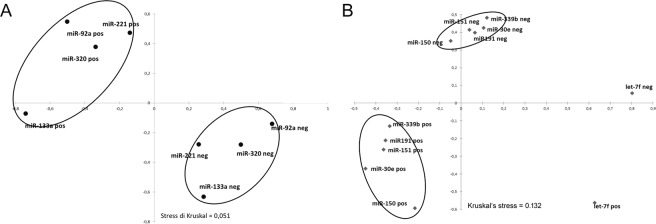
Multidimensional scaling plots comparing miRNA expression levels in the (**A**) blood serum and (**B**) vaginal fluids of *Brucella*-positive and negative buffaloes. For comparison to the seronegative buffaloes, seropositive buffaloes are grouped together.

Discriminant analysis was applied to further investigate the potential for improving diagnostic performance by analysing multiple DE-miRNAs. Statistical analysis was performed examining the weighted average relative quantification (RQ) values of the miRNAs with an AUC > 0.7 for serum blood (miR-320a and miR-92a) and AUC > 0.8 for vaginal fluid (miR-let-7f, miR-151, miR-30e, miR-191, miR-150 and miR-339b) (Fig. 4). Median expression levels in the blood sera were 0.649 (range, 0.229 to 1.639) and 0.542 (range, 0.188 to 1.889) and in the vaginal fluid 11.62 (range, −6.6 to 40.36) and −6.16 (range, −67.6 to 73.8) in *Brucella*-positive and *Brucella*-negative buffaloes, respectively (Fig. 4A,C). The predicted probability of being discriminated as positive from the logit model based on the two blood sera [logit = (0.6045 × expression level of miR-320a) + (0.4045 × expression level of miR-92a)] or the six vaginal fluid miRNAs [logit = (0.936 × expression level of let-7f) + (4.183 × expression level of miR-151) + (−13.777 × expression level of miR-339b) + (−21.946 × expression level of miR-30e) + (5.372 × expression level of miR-150) + (3.784 × expression level of miR-191)] was used to construct the ROC curves (Fig. 4C,D). The AUC for the combined blood serum miRNAs was 0.753 (95% CI 0.620–0.857) with a cut-off value of 0.90 and 80% sensitivity and 56.25% specificity. The AUC for the combined vaginal fluid miRNAs was 0.88 (95% CI 0.742–0.959) with a cut-off value of 0.023 and 95.45% sensitivity and 85% specificity. Clustering patterns were further visualized using multidimensional scaling plots (MDS) (Fig. 5). These plots generate distances between samples corresponding to the biological coefficient of variation between miRNAs in each sample. The MDS plots comparing seropositive and seronegative buffaloes showed distinct groupings of infected and control animals in both the blood serum and vaginal fluid samples, highlighting a clear miRNA expression distinction by infection type.

### miRNA target prediction and pathway enrichment

**Figure 6:**
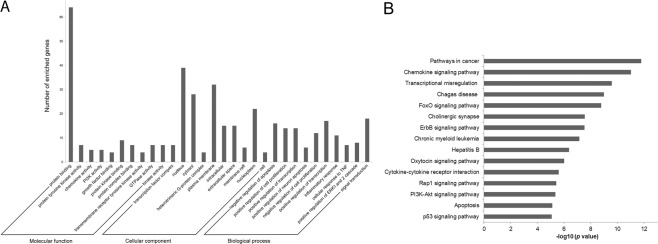
Target prediction and pathway enrichment. (**A**) GO annotation of genes regulated by identified DE-miRNAs. The target genes were annotated by DAVID at three levels: molecular function, cellular component and biological process. The top 10 significantly enriched items are shown. (**B**) Pathway enrichment analysis for genes regulated by DE-miRNAs. Genes regulated by DE-miRNAs were retrieved and enriched in KEGG using DAVID. The *P* value was negative 10-base log transformed. The top 15 enriched KEGG pathways are reported.

Predicted significant DE-miRNA targets were computationally fetched from the TargetScan database, and genes with cumulative weighted context++ scores (CWCS) < −0.4 were further analysed. mRNA was enriched using DAVID bioinformatics to explore the function and pathogenesis of these candidate biomarkers. Gene Ontology (GO) analysis was performed using DAVID at three levels: Molecular Function (MF), Cellular Component (CC) and Biological Process (BP). Figure 6A illustrates the top 10 items that were significantly enriched by target genes for each of the above GO levels. The enriched GO terms in MF mainly included protein binding and kinase activities. The CC items in which the predicted targets were involved were related to proteins involved in structure (nucleus and cytoplasm) and function (signal transduction and replication, such as G-protein complexes and transcription factors, respectively). Most GO BP items converged on regulating proliferation-apoptosis processes. KEGG pathway analysis was carried out on the whole targets of miRNA biomarkers using DAVID. Since altered immune responses are believed to contribute to the bacteria’s ability to hide and survive in the host, mRNA targets encoding for genes involved in immune pathways were enriched. The list of immune-related targets included 78 genes that were employed in further analyses. The top 15 significantly enriched KEGG pathways are outlined in Fig. 6B, with chemokine signalling, transcriptional misregulation, Chagas disease and FoxO signalling being the top pathways.

## Discussion

Although serum circulating miRNAs are some of the most promising clinical diagnostic or therapeutic biomarkers for diagnosing various diseases and stress disorders in both humans^33^ and animals^22,23^, their diagnostic potential in veterinary medicine remains to be fully explored.

To the best of the authors’ knowledge, the present study is the first to address the relationship between *Brucella* infection and miRNAs in dairy water buffaloes. We adopted a multistep approach, using miRNomics as a first step to identifying differentially expressed miRNAs in blood serum, then validated them in blood serum and vaginal fluid, which was shown to be a reliable source of microRNA for the first time. We selected vaginal fluid as a potential miRNA source because the reproductive system is the preferential target organ for *Brucella* infection. We found that (I) the levels of four miRNAs were significantly downregulated in the blood serum of seropositive buffaloes; (II) the levels of 10 miRNAs were significantly higher in the vaginal fluids of seropositive than in seronegative animals; (III) diagnostic accuracy for *B. abortus* was excellent (0.90 < AUC < 1) for two miRNAs (miR-151 and miR-30e) and good (0.80 < AUC < 0.90) for four miRNAs (miR-let-7f, miR-339b, miR-150 and miR-191); and (IV) the diagnostic accuracy of the combination of six differentially expressed miRNAs was good (AUC = 0.88) with 95.45% sensitivity and 85% specificity. We also demonstrated that miRNAs can be efficiently extracted from vaginal fluids.

Sequencing results provided a list of differentially abundant miRNAs but validation with RT-qPCR demonstrated that the differential expression rate in the serum between *Brucella*-seropositive and seronegative animals was not statistically significant. Therefore, the results suggested that miRNA expression levels were similar in the blood sera of seropositive and seronegative water buffaloes. Nevertheless, technical bias inherent in sequencing technologies may generate distorted results. A substantial distortion between miRNA levels in NGS data and true miRNA abundancy may occur using Illumina sequencing technology^34^. Because vaginal fluids may express the same miRNAs as in the serum during *B. abortus* infection^35^, these miRNAs were quantified, and the results are promising.

In addition to well-established tests, such as serological and microbiological tests, a molecular approach was used to broadly assess *Brucella* spp. infections^36–38^. MiRNAs regulate gene expression in many pathophysiological pathways, including those involved in microbial infection. Changes in host miRNA expression occur following infection with exclusively extracellular (*Helicobacter pylori*) or intracellular (*Salmonella enterica*) gram-negative bacteria, as well as in response to gram-positive bacteria (*Listeria monocytogenes*) and other pathogens (*Mycobacterium* and *Francisella* species)^17,39^. The infected cells modulate miRNAs, suppressing host immune responses and promoting microbial pathogenesis. For example, let-7 family miRNAs, which were upregulated in the present study, are involved in modulating two major cytokines, IL6 and IL10, which have opposite effects on the immune response: specifically IL6 promotes, whereas IL10 dampens the proinflammatory programme. The miR-150 family (both miR150 and miR-151 were downregulated in the present study) is involved in lymphocyte development and regulation; a recent study identified that serum circulating miR-150 is a general lymphocyte activation sensor and may serve as a biomarker for human lymphocyte activation in healthy and diseased conditions^40^. The miR-30 family, to which miR-30e belongs and which was downregulated in the present study, is involved in modulating host SUMOylation by downregulating UBC9, a protein involved in T cell development^41^. The results reported herein suggest that *B. abortus* may influence the immune responses of infected buffaloes by modulating the production and exocytosis of miRNAs that influence both innate and adaptive immune responses, potentially acting on phagocytic cells and lymphocytes as previously suggested for cattle^42^. Accurate screening tests are important for the success of brucellosis eradication programmes. This study suggests that vaginal DE-miRNA levels accurately differentiated *B. abortus*-infected buffaloes from controls. Many miRNAs aberrantly expressed mRNAs have been identified between *Brucella*-infected and noninfected buffaloes, and the relative expressions of DE-miRNAs, such as miR-151 and miR-30e, were used to discriminate seropositive *vs* seronegative animals. These miRNAs had high sensitivity (>94%) up to 100% for miR-151, but low specificity, which reached 85% when some of these were combined. These data showed that miRNAs collected from vaginal fluids may be suitable for use in screening because they can solve many problems that arise from other tests^14,15^. The sensitivity and specificity of the immunological tests used to routinely diagnose brucellosis in cattle have been summarized by several authors^16,43,44^ who believe that more than one test should be applied to serologically diagnose brucellosis in the field. The main issue concerns test sensitivity, which normally does not exceed 90%, whereas specificity can reach 100%. Quantifying a panel of miRNAs in the vaginal fluid increases sensitivity to 95% with an adequate 85% specificity. The authors believe that applying this miRNA panel may help to identify infected animals, particularly in extensive breeding systems where brucellosis is endemic or transmission is relatively low. Combining immunological and molecular tests may enable an ideal balance between specificity and sensitivity. Our results suggest that noninvasive molecular biomarkers may help to more accurately assess and monitor *Brucella* infections.

Gene Ontology analysis demonstrated that DE-miRNAs regulated mRNA coding for proteins involved in several molecular functions, cellular components and biological processes involved in *Brucella* pathogenesis. For example, these DE-miRNAs may target genes coding for G-proteins, plasma membranes and membrane rafts, which are essential for *Brucella* endocytosis, highlighted at the CC and MF levels. The pivotal role of these mechanisms was elucidated by Rossetti and colleagues^45^, who demonstrated that pathogens attaching to the cell surface induce a zipper-like mechanism for internalization, and binding activates the GTPases, promoting cytoskeleton reorganization and thus host cell membrane rearrangement. Inside cells, these pathogens reside in the *Brucella*-containing vacuole (BCV) and influence two other processes potentially targeted by DE-miRNAs: the inflammatory reaction, which is modulated by T4SS (type IV secretion system)^46^, and changes in host cell transcription processes^47^. PI3K (phosphatidylinositol 3-kinase) activities, highlighted by both GO and KEGG analysis, are involved in *Brucella* uptake^48^ and modulating host cell proinflammatory responses. PI3K promotes binding in TIR-containing proteins and subsequent TLR signalling activation. Sengupta and colleagues^49^ demonstrated that *Brucella* expressed the TIR-containing protein, Btp1, which competes with MyD88 and interferes with the TLR4/TLR2 cascade, dendritic cell maturation, and proinflammatory cytokine production. KEGG analysis identified genes and pathways related to cell apoptosis: proliferation and chemokine signalling. *B. abortus* infection does not lead to cell death^50^, and *B. suis* inhibits apoptosis induced by chemical stimuli in human macrophages^51^. The mechanisms underlying apoptosis inhibition, include modulating genes from the BCL2 pathway^51^, with both pro- and anti-apoptotic activities. The DE-miRNAs may modulate apoptosis-related genes, promoting cell survival by targeting prosurvival BCL2 proteins, such as BCL2 (B-cell lymphoma 2) and MCL1 (ML1 myeloid cell leukaemia 1), and prodeath proteins, such as BAK1 (Bcl-2 homologous antagonist/killed 1) and PUMA (p53-upregulated modulator of apoptosis). Apoptosis is an important innate immune mechanism that eliminates pathogen-infected cells; the possibility that *Brucella* may modulate host miRNAs regulating apoptotic signalling pathways might partially explain *Brucella’s* ability to promote propagation and evade host defences.

In conclusion, the present study identified for the first time DE-miRNAs in the blood serum and vaginal fluid of water buffaloes affected by *Brucella abortus* compared with healthy animals. These miRNAs were involved in regulating the transcriptions of genes related to the molecular pathogenesis of brucellosis. Moreover, we found that miR-let-7f, miR-151, miR-30e, miR-191, miR-150 and miR-339b extracted from vaginal fluids are potentially useful biomarkers of *Brucella* infection. Although this study provided new and important insights into brucellosis pathogenesis, further experiments involving more animals are required to validate the miRNAs’ potential use in diagnosing brucellosis. A better grasp of the mechanisms regulated by these miRNAs will be important for developing interventions such as designed therapy and vaccines.

## Materials and Methods

### Ethical statement

Samples were collected during routine disease testing for the national brucellosis eradication programme. The study design was approved by Campania Region DGR No. 352/2013 and DRD No. 603 dated 11/03/2014. All experiments were performed in accordance with the relevant guidelines and regulations.

### Animal husbandry, sample collection and oestrus phase classification

Blood and vaginal fluid samples were collected from 60 buffaloes (*Bubalus bubalis*) raised on 3 farms in the Caserta area in June and July 2016 during routine national brucellosis prophylaxis programme screening. Animals were housed in an open yard breeding system. Blood was collected by tail venepuncture in serum and Monovette EDTA tubes (Sarstedt Company, Nümbrecht, Germany) and centrifuged at 800 × g for 15 min. Serum and plasma were stored at −80 °C. To collect the vestibulovaginal fluid swabs, the buffaloes were restrained and a sterile swab (sterile swab on a wooden stick with a cotton tip in a polypropylene test tube (12 × 150 mm), FL Medical s.r.l. Unipersonale Padova, Italy) was introduced into the cranial vagina. The swab was exposed to the vestibule-vaginal fluid, pulled inside the pipette and stored at −80 °C.

Oestrous cycle stage was determined by progesterone (P4)^52–54^ and oestradiol (E2)^55^ plasma concentrations using radioimmune assays (RIA).

### Serological and bacteriological analysis

Serology for brucellosis was performed using the Rose-Bengal and complement fixation tests on all samples, as recommended by the World Organization for Animal Health (OIE) Manual of Diagnostic Tests and Vaccines for Terrestrial Animals 2017 (version adopted in May 2016)^56^. Microbiological analysis was performed as previously reported by O’Grady and colleagues^57^.

### MiRNA extraction, library preparation, and sequencing

Total RNA was extracted using the miRNeasy Serum/Plasma Kit (Qiagen, catalogue number 217184, Milan, Italy). Serum was thawed on ice and centrifuged at 3000 × g for 5 min at 4 °C. An aliquot of 200 μl per sample was transferred to a new tube, and RNA was extracted using miRNeasy Serum/Plasma Kits (Qiagen, catalogue number 217184, Milano, Italy) in accordance with the manufacturer’s instructions. Animals were divided into six pools of 5 by the presence or absence of *Brucella* infection, and the pools were sequenced.

Libraries were prepared using TruSeq SmallRNA Sample Prep kits (Illumina, San Diego, CA, USA) following the manufacturer’s instructions. Both RNA samples and final libraries were quantified using the Qubit 2.0 Fluorometer (Invitrogen, Carlsbad, CA, USA) and quality tested by the Agilent 2100 Bioanalyzer RNA Nano assay (Agilent Technologies, Santa Clara, CA, USA). Libraries were then processed with Illumina cBot for cluster generation on flowcells, following the manufacturer’s instructions and sequenced on single-end mode at the multiplexing level requested on HiSeq. 2500 (Illumina, San Diego, CA). The CASAVA 1.8.2 version of the Illumina pipeline was used to process raw data for both format conversion and demultiplexing^58^. The reads obtained were mapped to the *Bos taurus* database because the complete *Bubalus bubalis* genome is lacking.

### Validation by qPCR

Total RNA was extracted using the miRNeasy Serum/Plasma Kit (Qiagen, catalogue number 217184, Milano, Italy) as reported above. One millilitre of Qiazol (Qiagen) was added to an aliquot of 200 μl per sample. After incubation at room temperature for 5 min, 3.75 μl (25 fmol final concentration) of the exogenous synthetic spike-in control *Caenorhabditis elegans* miRNA cel-miR-39 (Qiagen, catalogue number 219610) was spiked into samples at the beginning of the extraction procedure. Reverse transcription was performed in 15 μl volume reactions using the TaqMan MicroRNA Reverse Transcription Kit (Applied Biosystems, catalogue number 4366596, Monza, Italy) using miRNA-specific stem-loop RT primers. The mix reactions contained 1.5 µl 10× miRNA RT buffer, 1 µl MultiScribe reverse transcriptase (50 U/µl), 0.30 µl 100 mM dNTP mix, 0.19 µl RNase Inhibitor (20 U/µl), 6 μl of custom RT primer pool and 3.01 μl of nuclease-free water. The custom RT primer pool was prepared by combining 10 µl of each 5× RT primer in a final volume of 1000 µl; the final concentration of each primer in the RT primer pool was 0.05× each. Three μl of serum RNA was added to each RT reaction. Each RT reaction mixture was incubated on ice for 5 min, 16 °C for 30 min, 42 °C for 30 min and 85 °C for 5 min.

To validate the sequencing results, the qPCR experiments were designed following MIQE guidelines^59^. Small RNA TaqMan assays were performed per the manufacturer’s instructions using the selected primer/probe assays (ThermoFisher Scientific, Monza, Italy) reported in Table 3. Quantitative reactions were performed in duplicate in scaled-down (12 μl) reaction volumes using 6 μl TaqMan 2X Universal Master Mix II (ThermoFisher Scientific, Monza, Italy, catalogue number 4440044), 0.6 μl miRNA specific TaqMan Assay 20× and 1μl of the RT product per reaction on the Eco Real-Time PCR detection system (Illumina, Milan, Italy). The standard cycling programme was 50 °C for 2 min, 95 °C for 10 min and 40 cycles at 95 °C for 15 sec and 60 °C for 60 sec. Data were normalized relative to the cel-miR-39 expression. MiRNA expression levels are presented as fold changes normalized to cel-miR-39 expression using the 2^−ΔΔCq^ formula. The significant miRNA targets were determined using the TargetScan database (http://www.targetscan.org/vert_71/), functional mRNA were enriched using DAVID bioinformatics resources (https://david.ncifcrf.gov/), and biological pathways in KEGG were examined for enrichment (http://www.genome.jp/kegg/).

**Table 3:** List of TaqMan probes (ThermoFisher Scientific, Monza, Italy) and assay IDs.

miRNA	Assay ID
cel-miR-39-3p	000200
hsa-let-7i	002221
hsa-miR-320a	002277
hsa-miR-92a	000431
hsa-miR-126-5p	000451
hsa-let-7f	000382
hsa-miR-151-3p	002254
hsa-miR-30e-5p	007791_mat
hsa-miR-150	006586_mat
hsa-miR-339b	241893_mat
hsa-miR-191	002299
mmu-miR-221	001134
hsa-miR-133a	002246

### Statistical analysis

Statistical analysis was performed using XLStat for Windows (Addinsoft, New York, U.S.A.) and MedCalc 14.0 (MedCalc Software bvba, Ostend, Belgium). Statistical significance was accepted at *P* < 0.05. Data were tested for normality and homogeneity of variance using the Kolmogorov-Smirnov and Levene tests, respectively. Because the data were not normally distributed, nonparametric statistical tests were applied. The Kruskal-Wallis test was used to assess differences in miRNA concentrations. *P* values were adjusted using the Bonferroni correction. Linear regression was used to investigate relationships between DE-miRNAs and oestrus phases. Receiver operating characteristic (ROC) analysis was performed to determine the diagnostic accuracy of targets that statistically differed between *Brucella*-positive and negative animals. The diagnostic values were calculated for miRNAs that showed significant differential expression in the buffalo blood. Distance matrices were processed by multidimensional scaling (MDS) to obtain a dimensionally reduced map of the miRNA coordinates. Linear regression was used to investigate relationships between miRNAs and the buffaloes’ ages. Spearman’s Rho test was performed to evaluate whether the expression levels of the various miRNAs were correlated.

## Supplementary information

Supplementary figure 1

## References

[1] Corbel MJ (1997). Brucellosis: an overview. Emerging Infect. Dis..

[2] Martirosyan A, Moreno E, Gorvel J-P (2011). An evolutionary strategy for a stealthy intracellular Brucella pathogen. Immunol. Rev..

[3] Carvalho Neta AV (2008). Modulation of the bovine trophoblastic innate immune response by Brucella abortus. Infect. Immun..

[4] McDermott J, Grace D, Zinsstag J (2013). Economics of brucellosis impact and control in low-income countries. Rev. - Off. Int. Epizoot..

[5] Singh BB, Dhand NK, Gill JPS (2015). Economic losses occurring due to brucellosis in Indian livestock populations. Prev. Vet. Med..

[6] Garofolo G (2017). Origins and global context of Brucella abortus in Italy. BMC Microbiol..

[7] Fosgate GT, Adesiyun AA, Hird DW, Hietala SK, Ryan J (2002). Isolation of Brucella abortus biovar 1 from cattle and water buffaloes on Trinidad. Vet. Rec..

[8] Megid J (2005). Isolation of Brucella abortus from cattle and water buffalo in Brazil. Vet. Rec..

[9] Ali S (2017). Seroprevalence and risk factors associated with bovine brucellosis in the Potohar Plateau, Pakistan. BMC Res Notes.

[10] Borriello G (2013). Link between geographical origin and occurrence of Brucella abortus biovars in cow and water buffalo herds. Appl. Environ. Microbiol..

[11] Wareth G (2014). Animal brucellosis in Egypt. J Infect Dev Ctries.

[12] Anderson TD, Cheville NF, Meador VP (1986). Pathogenesis of placentitis in the goat inoculated with Brucella abortus. II. Ultrastructural studies. Vet. Pathol..

[13] Anderson TD, Meador VP, Cheville NF (1986). Pathogenesis of placentitis in the goat inoculated with Brucella abortus. I. Gross and histologic lesions. Vet. Pathol..

[14] Capparelli R (2009). Heterogeneous shedding of Brucella abortus in milk and its effect on the control of animal brucellosis. J. Appl. Microbiol..

[15] Ducrotoy MJ, Conde-Álvarez R, Blasco JM, Moriyón I (2016). A review of the basis of the immunological diagnosis of ruminant brucellosis. Vet. Immunol. Immunopathol..

[16] Ducrotoy MJ, Muñoz PM, Conde-Álvarez R, Blasco JM, Moriyón I (2018). A systematic review of current immunological tests for the diagnosis of cattle brucellosis. Prev. Vet. Med..

[17] Ghai V, Wang K (2016). Recent progress toward the use of circulating microRNAs as clinical biomarkers. Arch. Toxicol..

[18] Eulalio A, Schulte L, Vogel J (2012). The mammalian microRNA response to bacterial infections. RNA Biol.

[19] Turchinovich A, Weiz L, Langheinz A, Burwinkel B (2011). Characterization of extracellular circulating microRNA. Nucleic Acids Res..

[20] Markopoulos GS (2017). A step-by-step microRNA guide to cancer development and metastasis. Cell Oncol (Dordr).

[21] Hawleyeul ZCE, Campos-Melo D, Droppelmann CA, Strong MJ (2017). MotomiRs: miRNAs in Motor Neuron Function and Disease. Front Mol Neurosci.

[22] Lecchi C (2016). Circulating extracellular miR-22, miR-155, and miR-365 as candidate biomarkers to assess transport-related stress in turkeys. Animal.

[23] Lecchi C (2018). Circulating miR-23b-3p, miR-145-5p and miR-200b-3p are potential biomarkers to monitor acute pain associated with laminitis in horses. Animal.

[24] Dong H (2017). Circulating MicroRNAs As Potential Biomarkers for Veterinary Infectious Diseases. Front Vet Sci.

[25] Correia CN (2017). Circulating microRNAs as Potential Biomarkers of Infectious Disease. Front Immunol.

[26] Gu H (2017). Salmonella produce microRNA-like RNA fragment Sal-1 in the infected cells to facilitate intracellular survival. Sci Rep.

[27] Wang B (2016). Comprehensive identification and profiling of Nile tilapia (Oreochromis niloticus) microRNAs response to Streptococcus agalactiae infection through high-throughput sequencing. Fish Shellfish Immunol..

[28] Sengar GS (2018). Identification of differentially expressed microRNAs in Sahiwal (Bos indicus) breed of cattle during thermal stress. Cell Stress Chaperones.

[29] Cui B (2017). Brucella Omp25 Upregulates miR-155, miR-21-5p, and miR-23b to Inhibit Interleukin-12 Production via Modulation of Programmed Death-1 Signaling in Human Monocyte/Macrophages. Front Immunol.

[30] Rong H (2017). CD14 gene silencing alters the microRNA expression profile of RAW264.7 cells stimulated by Brucella melitensis infection. Innate Immun.

[31] Zheng K (2012). MicroRNA expression profile in RAW264.7 cells in response to Brucella melitensis infection. Int. J. Biol. Sci..

[32] Carvalho Neta AV (2010). Pathogenesis of bovine brucellosis. Vet. J..

[33] Petrovic N, Ergün S, Isenovic ER (2017). Levels of MicroRNA Heterogeneity in Cancer Biology. Mol Diagn Ther.

[34] Baroin-Tourancheau A, Benigni X, Doubi-Kadmiri S, Taouis M, Amar L (2016). Lessons from microRNA Sequencing Using Illumina Technology. Advances in Bioscience and Biotechnology.

[35] El-Mogy M (2018). Diversity and signature of small RNA in different bodily fluids using next generation sequencing. BMC Genomics.

[36] Sergueev, K. V., Filippov, A. A. & Nikolich, M. P. Highly Sensitive Bacteriophage-Based Detection of Brucella abortus in Mixed Culture and Spiked Blood. *Viruses* **9** (2017). 10.3390/v9060144 PMC5490821 28604602

[37] Dos Santos LS (2017). Detection of Brucella sp. infection through serological, microbiological, and molecular methods applied to buffaloes in Maranhão State, Brazil. Trop Anim Health Prod.

[38] Kim J-Y (2016). Differential diagnosis of Brucella abortus by real-time PCR based on a single-nucleotide polymorphisms. J. Vet. Med. Sci..

[39] Das K, Garnica O, Dhandayuthapani S (2016). Modulation of Host miRNAs by Intracellular Bacterial Pathogens. Front Cell Infect Microbiol.

[40] de Candia P, Torri A, Pagani M, Abrignani S (2014). Serum microRNAs as Biomarkers of Human Lymphocyte Activation in Health and Disease. Front Immunol.

[41] Wang A (2017). Ubc9 Is Required for Positive Selection and Late-Stage Maturation of Thymocytes. J. Immunol..

[42] Lawless N, Vegh P, O’Farrelly C, Lynn DJ (2014). The Role of microRNAs in Bovine Infection and Immunity. Front Immunol.

[43] Abernethy DA (2012). Field trial of six serological tests for bovine brucellosis. Vet. J..

[44] Gall D, Nielsen K (2004). Serological diagnosis of bovine brucellosis: a review of test performance and cost comparison. Rev. - Off. Int. Epizoot..

[45] Rossetti CA, Drake KL, Adams LG (2012). Transcriptome analysis of HeLa cells response to Brucella melitensis infection: a molecular approach to understand the role of the mucosal epithelium in the onset of the Brucella pathogenesis. Microbes Infect..

[46] de Figueiredo P, Ficht TA, Rice-Ficht A, Rossetti CA, Adams LG (2015). Pathogenesis and immunobiology of brucellosis: review of Brucella-host interactions. Am. J. Pathol..

[47] Lamontagne J (2009). Intracellular adaptation of Brucella abortus. J. Proteome Res..

[48] Qin Q-M (2008). RNAi screen of endoplasmic reticulum-associated host factors reveals a role for IRE1alpha in supporting Brucella replication. PLoS Pathog..

[49] Sengupta D (2010). Subversion of innate immune responses by Brucella through the targeted degradation of the TLR signaling adapter, MAL. J. Immunol..

[50] Chaves-Olarte E (2002). Activation of Rho and Rab GTPases dissociates Brucella abortus internalization from intracellular trafficking. Cell. Microbiol..

[51] Gross A, Terraza A, Ouahrani-Bettache S, Liautard JP, Dornand J (2000). *In vitro* Brucella suis infection prevents the programmed cell death of human monocytic cells. Infect. Immun..

[52] Vecchio D (2012). Corpus luteum development and function and relationship to pregnancy during the breeding season in the Mediterranean buffalo. Theriogenology.

[53] Niswender GD (1973). Influence of the site of conjugation on the specificity of antibodies to progesterone. Steroids.

[54] Skaggs CL, Able BV, Stevenson JS (1986). Pulsatile or continuous infusion of luteinizing hormone-releasing hormone and hormonal concentrations in prepubertal beef heifers. J. Anim. Sci..

[55] Campanile G (2010). Growth, metabolic status and ovarian function in buffalo (Bubalus bubalis) heifers fed a low energy or high energy diet. Anim. Reprod. Sci..

[56] OIE. Manual of Diagnostic Tests and Vaccines for Terrestrial Animals 2017, http://www.oie.int/standard-setting/terrestrial-manual/access-online/ (2017).

[57] O’Grady D (2014). A comparative assessment of culture and serology in the diagnosis of brucellosis in dairy cattle. Vet. J..

[58] Baldan F (2016). Identification of tumorigenesis-related mRNAs associated with RNA-binding protein HuR in thyroid cancer cells. Oncotarget.

[59] Bustin SA (2009). The MIQE guidelines: minimum information for publication of quantitative real-time PCR experiments. Clin. Chem..

