# Supplementary material for: Characterization of circulating miRNA signature in water buffaloes (Bubalus bubalis) during Brucella abortus infection and evaluation as potential biomarkers for non-invasive diagnosis in vaginal fluid
Source: Sci Rep. 2019 Feb 13;9:1945. doi: 10.1038/s41598-018-38365-x (PMC6374377; doi:10.1038/s41598-018-38365-x)
Supplement: Supplementary file 1 — Supplementary figure 1 [file 41598_2018_38365_MOESM1_ESM.pdf]

Supplementary figure 1

Characterization of circulating miRNA signature in water buffaloes (*Bubalus bubalis*) during *Brucella abortus* infection and evaluation as potential biomarkers for non-invasive diagnosis in vaginal fluid

Cristina Lecchi[1*], Carlotta Catozzi[1], Valentina Zamarian[1], Gaia Poggi[4], Giorgia Borriello[2], Alessandra Martucciello[2,3], Domenico Vecchio[2,3], Esterina DeCarlo[2,3], Giorgio Galiero[2], Fabrizio Ceciliani[1].

(1) *Università degli Studi di Milano, Dipartimento di Medicina Veterinaria, Milano, 20133, Italy*

(2) *Istituto Zooprofilattico Sperimentale del Mezzogiorno, Portici, 80055, Italy*

(3) *Centro di Referenza Nazionale sull'igiene e le tecnologie dell'allevamento e delle produzioni bufaline, Salerno, 84132, Italy*

(4) *Diabetes Research Institute (DRI), San Raffaele Scientific Institute, Milano, 20132, Italy*

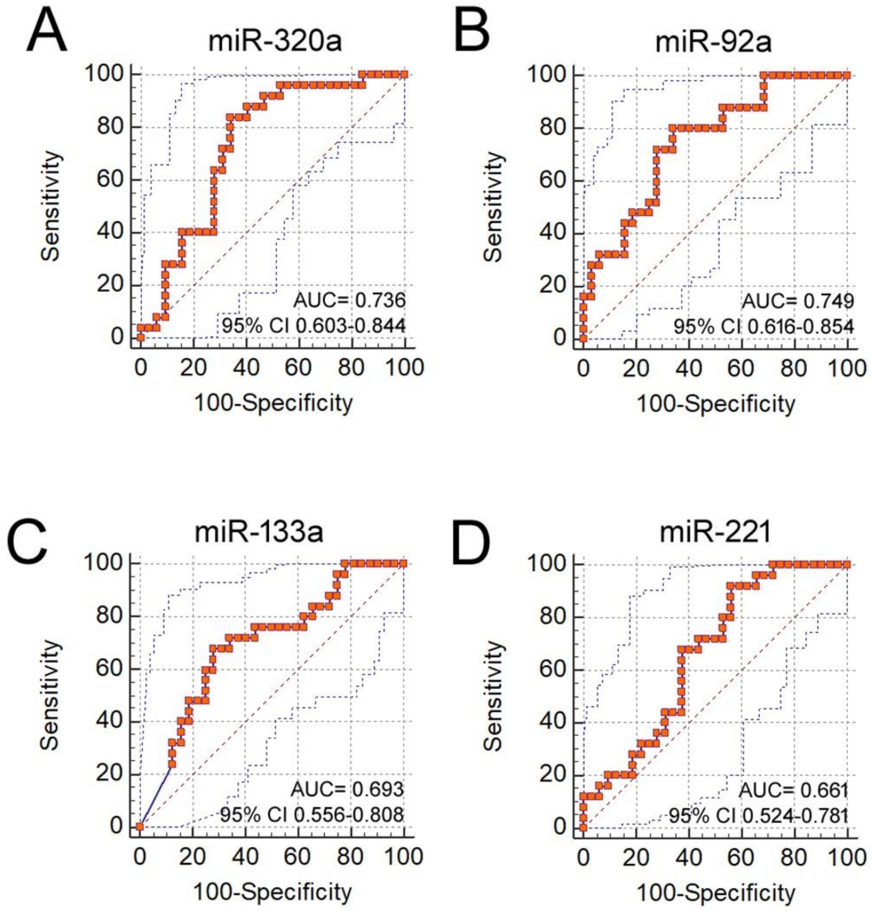

Supplementary figure 1. Receiver-operator characteristics (ROC) curve analysis of DE- miRNAs in blood. AUC, area under the curve; CI, confidence interval.
